# Study of Molecular Genetic Characteristics of Influenza A(H1N1) Viruses Identified During Monitoring in Kazakhstan (2023–2026)

**DOI:** 10.3390/v18101087

**Published:** 2026-10-01

**Authors:** Nailya Klivleyeva, Tatyana Glebova, Assem Baimukhametova, Kobey Karamendin, Nurbol Saktaganov, Nuray Ongarbayeva, Mereke Kalkozhayeva, Indira Ibragimova, Aknur Mutaliyeva, Altynay Gabiden, Richard Webby

**Affiliations:** 1 The Research and Production Center for Microbiology and Virology, Almaty 050010, Kazakhstannuray.syrlybay@gmail.com (N.O.);; 2 Postgraduate Cardiology Course, Kazakhstan-Russian Medical University, Almaty 050000, Kazakhstan; 3 National Center of Public Health Care, The Ministry of Health of the Republic of Kazakhstan, Almaty 050008, Kazakhstan; mutaliyeva.a.s@gmail.com (A.M.); altinai_s@mail.ru (A.G.); 4 Department of Infectious Diseases, St. Jude Children’s Research Hospital, Memphis, TN 38105-3678, USA

**Keywords:** virus, influenza, A(H1N1)pdm09, real-time PCR, mutations, neuraminidase inhibitors, oseltamivir, zanamivir, epidemic season, genetic clade 6B.1A.5a.2a

## Abstract

Background/Objectives: Seasonal influenza threatens global public health through rapid mutations. This study evaluated the prevalence and molecular-genetic characteristics of seasonal influenza viruses circulating in Kazakhstan during the 2023–2026 epidemic seasons. Methods: Nasopharyngeal swabs (*n* = 1756) and serum samples (*n* = 718) were screened using RT-qPCR, HI, and ELISA assays. Selected A(H1N1)pdm09 isolates underwent full-length HA and NA gene next-generation sequencing, followed by phylogenetic and phenotypic analyses. Results: Influenza A(H3N2) strains dominated in Kazakhstan during the 2023–2024 and 2025–2026 seasons. Conversely, the 2024–2025 season showed a shifting landscape, characterized by co-circulation of influenza B and A(H1N1)pdm09 strains belonging to genetic clade 6B.1A.5a.2a (subclades C.1.9 and C.1.9.3). Mutational mapping revealed ongoing in situ microevolution adjacent to the receptor-binding site (HA-R222K). Genomic screening of the NA gene identified critical I223R and S247N mutations traditionally linked to resistance. In silico profiling predicted that while the majority maintained baseline sensitivity, the solitary A/Shymkent/NRL-1999/2024 isolate harboring the I223R substitution exhibited a multidrug-resistant genotype to oseltamivir, peramivir, and zanamivir. Conclusions: These findings demonstrate dynamic inter-seasonal etiological shifts and the predicted emergence of an independent resistant lineage (NA-I223R). This underscores the necessity for continuous, expanded genomic surveillance of respiratory pathogens across Kazakhstan.

## 1. Introduction

Acute respiratory viral infections (ARVIs) remain a primary driver of seasonal hospitalizations, particularly among pediatric populations. These infections can induce severe clinical complications, including otitis media, bronchiolitis, and pneumonia, or exacerbate underlying chronic conditions such as asthma and chronic obstructive pulmonary disease. In severe cases, progression to respiratory failure, acute respiratory distress syndrome, cardiovascular insufficiency, and central nervous system damage may occur [1,2]. Among the diverse spectrum of respiratory pathogens, influenza viruses, respiratory syncytial virus, rhinoviruses, and coronaviruses present the highest clinical risks. For instance, respiratory syncytial virus serves as the leading cause of severe lower respiratory tract pathology (bronchiolitis) and is associated with substantial mortality rates among infants during their first year of life [3,4,5,6].

Influenza viruses exhibit exceptional evolutionary plasticity. Owing to a segmented, negative-sense RNA genome that lacks proofreading and error-correction mechanisms during replication, nucleotide mutations continuously accumulate. Antigenic drift and antigenic shift operate as perpetual evolutionary forces, causing influenza viruses to circulate within host populations as dynamic quasispecies—genetically diverse yet closely related mutant swarms. Within ancestral natural reservoirs, such as waterfowl and bats, influenza A viruses (IAVs) display minimal evolutionary pressure and typically cause asymptomatic infections. However, cross-species transmission events often trigger rapid adaptive evolution, culminating in devastating epidemics and pandemics across poultry, lower mammals, and human populations. This interspecies spillover is fundamentally driven by antigenic shift—the reassortment of viral gene segments within a single host, such as swine, simultaneously co-infected with distinct avian and human influenza strains [7,8,9,10].

Following pandemic introduction, influenza viruses establish sustained circulation within the human population, causing predictable seasonal surges in morbidity while undergoing continuous adaptive changes. This progressive antigenic drift enables the virus to evade host immune memory and reinfect individuals previously exposed to ancestral variants, yielding global pandemics every 10 to 50 years [11,12,13]. Since the dawn of the 20th century, global public health has recorded at least five high-mortality pandemics, four of which were etiologically linked to IAVs: the 1918 Spanish flu (H1N1), the 1957 Asian flu (H2N2), the 1968 Hong Kong flu (H3N2), and the 2009 swine flu (H1N1pdm09), alongside the 2019 COVID-19 pandemic caused by SARS-CoV-2. In the post-pandemic phase, these viruses transition into seasonal pathogens, collectively responsible for an estimated 3 to 5 million severe clinical cases and 290,000 to 650,000 deaths globally each year. In Kazakhstan, robust epidemiological surveillance systems register between 600,000 and 1.0 million laboratory-confirmed or clinically verified ARVI cases annually [14,15].

The lineage of the human A(H1N1) influenza virus tracks back to the historic 1918 pandemic, when an avian-ancestor virus crossed the species barrier into both humans and swine. Over the subsequent century, complex evolutionary trajectories and reassortment events shaped the viral architecture, culminating in the emergence of the modern triple-reassortant A(H1N1)pdm09 virus of swine origin [16,17,18]. During the 2009 pandemic, this novel strain caused substantial global mortality, with the estimated death toll reaching up to 575,000 individuals worldwide [19].

To counter this ongoing threat and ensure the selection of immunogenically matched candidate vaccine viruses (CVVs) for upcoming epidemic seasons, the World Health Organization (WHO) coordinates continuous global influenza surveillance. In Kazakhstan, as part of this international network, systematic genomic monitoring is actively conducted. This continuous surveillance is essential for defining dominant genetic lineages down to specific clades and subclades, tracing evolutionary trends, and tracking the introduction, expansion, or extinction of distinct viral variants within regional and global circulation networks.

## 2. Materials and Methods

### 2.1. Clinical Samples Collection

During the 2023–2026 epidemic seasons, a total of 1756 nasopharyngeal swabs and 718 blood serum samples were collected in healthcare facilities across Kazakhstan. Biological samples were obtained by healthcare workers from patients with acute respiratory viral infection (ARVI) symptoms (born between 1941 and 2026; 55.01% women, *n* = 966; 44.99% men, *n* = 790). The age distribution of the samples included children under 17 (60.31%), people of working age (28.76%), and the elderly (10.93%). The most common primary clinical diagnosis was ARVI (94.31%), followed by bronchitis (2.90%) and pneumonia (2.79%). The geographic distribution of the collected specimens was unevenly distributed across the country (Table 1).

The study was conducted in strict accordance with the principles of the Declaration of Helsinki and received formal approval from the Research Ethics Committee of the Scientific Production Center of Microbiology and Virology, Almaty, Kazakhstan (Protocol No. 19, dated 1 April 2025). All biological samples were processed in compliance with established ethical standards to guarantee absolute patient data confidentiality and anonymity.

Sample collection and primary handling were performed in adherence to the WHO guidelines [20]. Upon collection, each nasopharyngeal swab was immediately immersed in 2 mL of viral transport medium (Minimal Essential Medium; Gibco, Invitrogen, Grand Island, NY, USA) and transported under refrigerated conditions (4 °C). In the laboratory, specimens were vortexed for 15 s, centrifuged at 1000× *g* for 10 min at 4 °C, and the resulting supernatants were aliquoted and stored at −80 °C until further molecular analysis. Serum was separated from whole blood samples via centrifugation at 400× *g* for 15 min and stored at −20 °C.

### 2.2. Typing, Detection, and Sequencing of Influenza a Virus

#### 2.2.1. RNA Extraction and RT-qPCR Assay

Viral RNA was extracted from 140 µL of clinical sample supernatants using the commercial QIAamp Viral RNA Mini Kit (Qiagen, Hilden, Germany) strictly following the manufacturer’s operational manual.

Molecular genetic screening and typing of the isolates were performed via real-time reverse transcription-polymerase chain reaction (RT-qPCR). Amplification was carried out on a Rotor-Gene Q 6plex instrument (QIAGEN, Hilden, Germany) using standardized protocols adapted from the US Centers for Disease Control and Prevention (CDC) and the WHO. The qualitative detection of influenza A and B viruses, alongside the specific subtyping of seasonal influenza A variants (H1N1pdm09 and H3N2), was accomplished using a multiplex RT-qPCR configuration. The definitive sequences of the oligonucleotide primers and hydrolysis (dual-labeled) TaqMan probes utilized in this study are summarized in Table 2.

To ensure validation and cross-contamination control during nucleic acid extraction and thermal amplification, positive control samples containing inactivated reference influenza virus strains were included in each independent operational run. A no-template control (NTC) utilizing nuclease-free deionized water was integrated into every assay to exclude reagent or environmental contamination. Sampling adequacy, RNA extraction efficiency, and potential PCR inhibition were systematically monitored via the amplification of the human endogenous RNase P (RP) housekeeping gene.

The optimized single-step RT-qPCR thermocycling profile was executed as follows:

Reverse transcription: 50 °C for 15 min;

Polymerase activation and initial denaturation: 95 °C for 5 min;

Multiplex amplification (45 cycles): denaturation at 95 °C for 10 s, followed by a combined annealing/extension step at 55 °C for 20 s.

Real-time fluorescence signals were recorded during the combined annealing/extension phase across three discrete optical channels simultaneously: Green (FAM), Yellow (HEX/VIC), and Orange (ROX). Upon successful detection and threshold cycle (*C*_t_) verification of influenza virus RNA, target samples were directed to full-length sequencing of the hemagglutinin (HA) and neuraminidase (NA) genes for subsequent phylogenetic assessment.

#### 2.2.2. Detection of Antibodies in Serological Studies

To eliminate non-specific hemagglutination inhibitors, serum samples were treated with a commercial receptor-destroying enzyme (RDE) at a 3:1 (*v*/*v*) ratio (enzyme-to-serum) with a working dilution of 1:50, followed by incubation for 18 h at 37 °C. Prior to serological evaluation, the treated sera were diluted with phosphate-buffered saline (PBS) and heat-inactivated at 56 °C for 30 min.

The presence and titers of specific antibodies targeting influenza virus HA were determined using the hemagglutination inhibition (HI) assay and enzyme-linked immunosorbent assay (ELISA) in strict accordance with the WHO recommendations [20,21]. The HI assays were performed using commercial diagnostic antigen kits representing the following reference strains: A/Michigan/45/2015 (H1N1)pdm09, A/Singapore/INFIMH-16-0019/2016 (H3N2), B/Phuket/3073/2013 (Yamagata lineage), and B/Colorado/06/2017 (Victoria lineage). These antigens, along with the ELISA kits utilized for the qualitative and quantitative detection of antibodies against influenza A(H1N1), A(H3N2), and influenza B viruses, were manufactured by the Smorodintsev Research Institute of Influenza (St. Petersburg, Russia).

#### 2.2.3. Whole-Genome Amplification and Next-Generation Sequencing

Amplification of the full-length influenza virus genome was carried out via a single-step reverse transcription polymerase chain reaction (RT-PCR) using the commercial BioMaster RT-PCR-RV (2×) kit (Biolabmix, Koltsovo, Russia) on a CFX96 Touch Real-Time PCR Detection System (Bio-Rad, Hercules, CA, USA) according to modified universal influenza protocols [22,23]. The resulting whole-genome amplicons were verified by horizontal electrophoresis in a 2% agarose gel prepared in 1× TAE (Tris-acetate-EDTA) buffer and visualized using a Doc-Print CX3 gel documentation system equipped with an HC-40 hood and a 312 nm UV transilluminator (Vilber, Collégien, France).

Libraries for next-generation sequencing (NGS) were constructed using the Illumina DNA Prep kit (Illumina, San Diego, CA, USA) following the manufacturer’s operational manual. Sample indexing was performed using the IDT^®^ Illumina^®^ DNA/RNA Unique Dual (UD) Indexes (Sets A/B/C/D). Prior to library preparation, the whole-genome PCR products were purified using AMPure XP magnetic beads (Beckman Coulter, Brea, CA, USA).

The concentration of double-stranded DNA (dsDNA) in the purified amplicons was quantified using a Qubit 4 fluorometer (Thermo Fisher Scientific, Waltham, MA, USA) and the Qubit dsDNA HS (High Sensitivity) Assay Kit (Life Technologies, Carlsbad, CA, USA). Purified cDNA samples were normalized and diluted to a final master concentration of 0.2 ng/µL before library synthesis. High-throughput sequencing was executed utilizing the Illumina NextSeq 100-cycle P2 reagent kit on an Illumina NextSeq 2000 sequencing platform (Illumina, San Diego, CA, USA).

### 2.3. Sequence Processing and Phylogenetic Analysis

Primary processing and management of the raw sequencing data were conducted on a remote Linux-based computing server accessed via the MobaXterm software platform (v23.4; Mobatek, Toulouse, France). Multiple sequence alignments of the full-length HA and NA genes were performed using the ClustalW algorithm implemented within the MegAlign module of the Lasergene software suite (v15.0; DNASTAR, Madison, WI, USA). The generated sequences were compared against international reference, seasonal, and vaccine strains retrieved from the GISAID (https://gisaid.org) and GenBank (nih.gov) public databases.

Lineage classification, genetic clade determination, and subclade assignment were accomplished using the Nextclade web tool (nextstrain.org). Genetic variations, including amino acid substitutions, insertions, deletions, nucleotide deviations, and potential N- and O-glycosylation sites, were systematically mapped using Nextclade v3.18.1 [24].

Phylogenetic reconstruction was executed via the Maximum Likelihood (ML) method using the MEGA12 software suite (version 12.1.2) [25]. The optimal nucleotide substitution models were determined based on the lowest Bayesian Information Criterion (BIC) scores. The Hasegawa–Kishino–Yano model with a discrete gamma distribution of rates (HKY + G) was selected as the best-fit model for the HA gene phylogenetic reconstruction, with the final dataset comprising 41 sequences spanning 1650 nucleotide positions [26]. For the NA gene, the Tamura-3-parameter model with a discrete gamma distribution (T92 + G) was applied to a final dataset containing 14 sequences spanning 1406 nucleotide positions [27]. To account for evolutionary rate heterogeneity among sites, a discrete five-category gamma distribution (+G) was modeled, utilizing a shape parameter of (α = 0.3182) for HA and (α = 0.3193) for NA. The topological robustness and branch reliability of the reconstructed trees were statistically evaluated using bootstrap analysis with 1000 pseudo-replicates [28,29]. Two different reference viruses were used in the bioinformatics data processing. Assignment to genetic clades and subclades (6B.1A.5a.2a and proposed lineages C.1.9/C.1.9.3) was performed using the current WHO-recommended reference strain A/Lisboa/188/2023, using the Nextclade and GISAID alignment algorithms. In parallel, the A/Sydney/5/2021(H1N1) strain was used as the initial (ancestral) reference for descriptive analysis and calculation of the cumulative number of nonsynonymous amino acid substitutions over successive epidemic seasons.

### 2.4. Statistical Analysis

To analyze the distribution shifts and positive PCR rates across three consecutive influenza seasons, the Chi-square (χ^2^) test of independence was initially performed. For subsequent pairwise inter-season comparisons, Fisher’s exact test was utilized to accommodate low cell counts within the generated contingency tables. To control the family-wise error rate arising from multiple testing, the *p*-values for all post hoc pairwise comparisons were systematically adjusted using the Holm–Bonferroni correction method. All statistical computations were executed using the Python programming language (v3.10) utilizing the SciPy library suite (v1.11). Statistical significance for all tests was established at an adjusted threshold of *p* < 0.05.

## 3. Results

### 3.1. Influenza Surveillance Dynamics and Subtype Distribution

Molecular genetic screening via RT-qPCR revealed that influenza virus genetic material was detected in 146 out of the 1756 analyzed nasopharyngeal swabs, yielding an overall baseline positivity rate of 8.31% (Table 3). Within this positive cohort, influenza A viruses (IAVs) were detected in 125 samples (7.12% of the total sample), while influenza B virus was identified in 21 samples (1.20%). Within the IAV-positive structure, influenza A(H1N1)pdm09 virus RNA was detected in 16 samples (0.91% of the total sample), and influenza A(H3N2) virus RNA was confirmed in 65 samples (3.70%). In 44 IAV-positive specimens (2.51% of the total sample), the specific subtype could not be determined due to low viral load or technical constraints. Cumulatively across the multi-year study period, the A(H3N2) subtype operated as the predominant causative agent among circulating influenza viruses. However, a profound etiological shift occurred during the intermediate 2024–2025 season, wherein influenza A(H1N1)pdm09 emerged as the primary etiological agent, accounting for 1.73% of the total respiratory disease burden. Statistical analysis comparing the observed annual virus detection rates with expected baseline values indicated that the inter-seasonal variations for H1N1, H3N2, type B, and non-typeable IAVs did not reach statistical significance when evaluated globally across the entire multi-year structure.

Consistent with international trends tracked via the Global Influenza Programme’s web-based platform (FluNet, who.int, accessed on 1 May 2026), influenza circulation in Kazakhstan from 2023 to 2026 exhibited strict seasonal epidemic patterns. National surveillance database extraction showed that out of 43,109 specimens tested from patients presenting with influenza-like illnesses, influenza types A and B were verified in 7.19% (3101/43,109) of the cases [30]. The respective overall shares for influenza A(H1N1)pdm09, A(H3N2), and influenza B viruses were 0.57% (247/43,109), 5.92% (2554/43,109), and 0.69% (297/43,109).

To assess the epidemiological contribution of influenza viruses to the complex polymicrobial picture of circulating ARIs, an analysis of the detection rate of pathogens was performed taking into account age stratification. The study sample (*N* = 1756 respiratory material samples) was divided into four separate, socio-demographically significant age categories: young children (0–4 years; *n* = 416), school-age children and adolescents (5–17 years; *n* = 643), young and middle-aged adults (18–65 years; *n* = 505), and elderly people (65 years and older; *n* = 192). Stratification revealed clear age differences in the overall influenza detection rates (including all confirmed cases of influenza types A and B). The highest overall detection rate was recorded in the group of economically active adults aged 18–65 years and amounted to 10.30% (52/505); followed by the elderly group (≥65 years) with a rate of 9.38% (18/192) and the young children group (0–4 years) with a rate of 9.13% (38/416). In contrast, the lowest overall detection rate was observed among school-age children and adolescents (5–17 years)—5.91% (38/643). To assess the statistical significance of the obtained results, the Pearson chi-square test was applied to the influenza incidence matrix, taking into account age stratification; the analysis showed that the observed differences between the identified age groups are statistically significant (χ^2^ = 8.132; df = 3; *p* = 0.043; Table 4).

A detailed analysis of the distribution of the A/H1N1pdm09 subtype showed that the peak of its detection also occurred in the adult age group (18–65 years) and amounted to 1.78% (9/505). At the same time, the prevalence of the A/H1N1 virus (taking into account typing) remained low and relatively uniform in the other groups: 0.72% (3/416) in the 0–4 age category, 0.52% (1/192) among the elderly, and 0.47% (3/643) among school-age children. Analysis of the distribution of the detection rate of A/H1N1 using the Pearson chi-square test showed that these differences between the groups did not reach the level of statistical significance (χ^2^ = 6.142; df = 3; *p* = 0.105).

According to international trends monitored through the web platform of the Global Influenza Program (FluNet), influenza virus circulation in Kazakhstan in the period from 2023 to 2026 was characterized by pronounced seasonal epidemic dynamics. Analysis of data from the national epidemiological surveillance database showed that out of 43,109 samples obtained from patients with influenza-like illnesses, influenza viruses of types A and B were detected in 7.19% of cases (3101 out of 43,109) [30]. The proportion of influenza viruses A(H1N1)pdm09, A(H3N2) and B was 0.57% (247/43,109), 5.92% (2554/43,109) and 0.69% (297/43,109), respectively.

**Figure 1:**
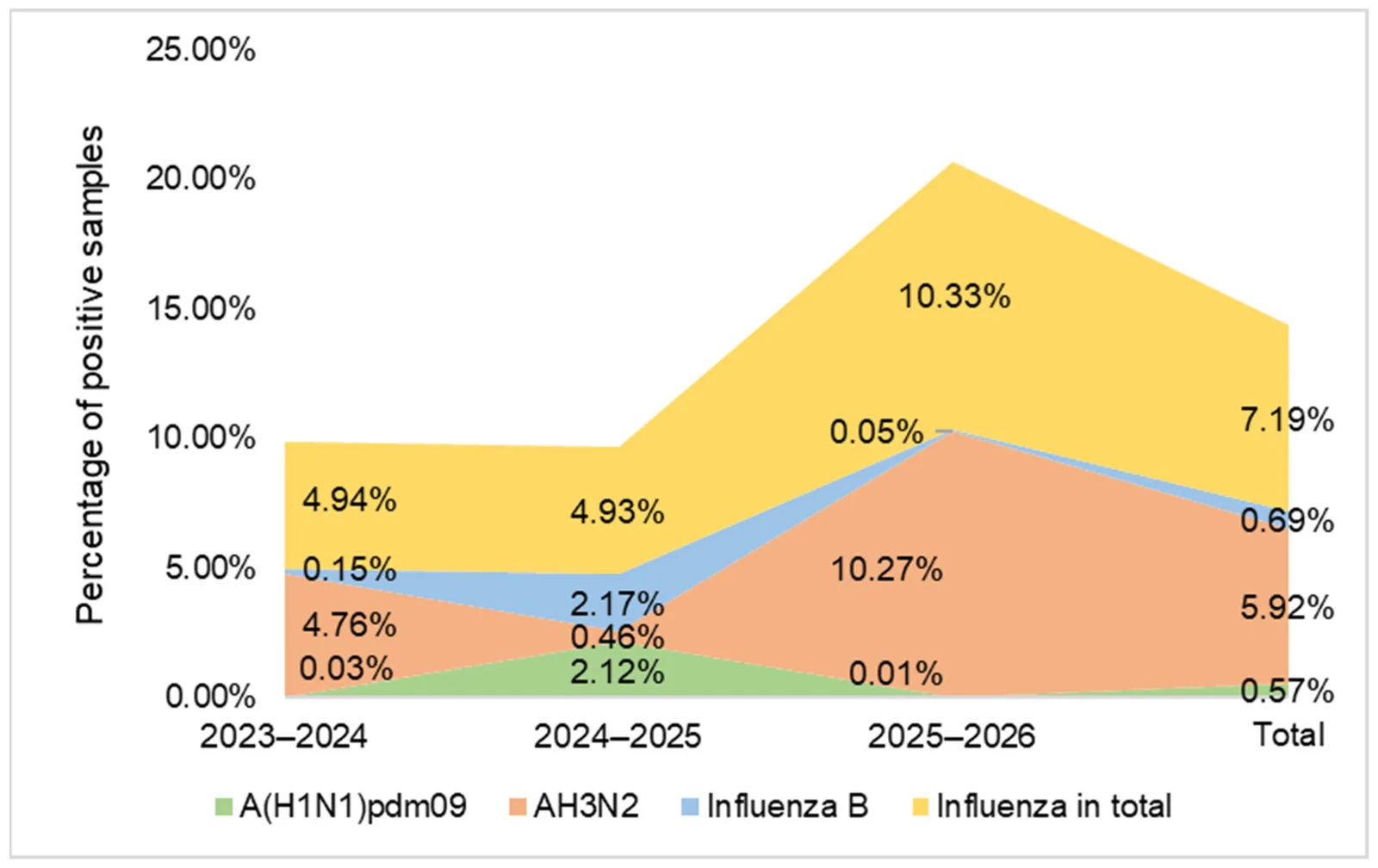
Influenza laboratory surveilance information. Data of World Health Organization for Kazakhstan in 2023–2026. Virus detection by subtype reported to FluNet [30].

The A(H3N2) subtype demonstrated unprecedented dominance during the 2023–2024 and 2025–2026 epidemic boundaries [31]. For example, during the 2023–2024 cycle, 13,654 sentinel and non-sentinel ILI samples were processed, yielding only four confirmed cases of influenza A(H1N1)pdm09, which translated to a marginal positivity rate of 0.59% (4/675) among validated influenza-positive cases. Conversely, the 2024–2025 epidemic window was marked by a complete restructuring of the viral landscape, where influenza A(H1N1)pdm09 dominated alongside influenza B variants. Out of 11,426 tested samples during this period, A(H1N1)pdm09 was confirmed in 242 cases, representing a sharp, statistically significant surge to a 42.98% (242/563) positivity rate among typed influenza isolates (Figure 1).

**Figure 2:**
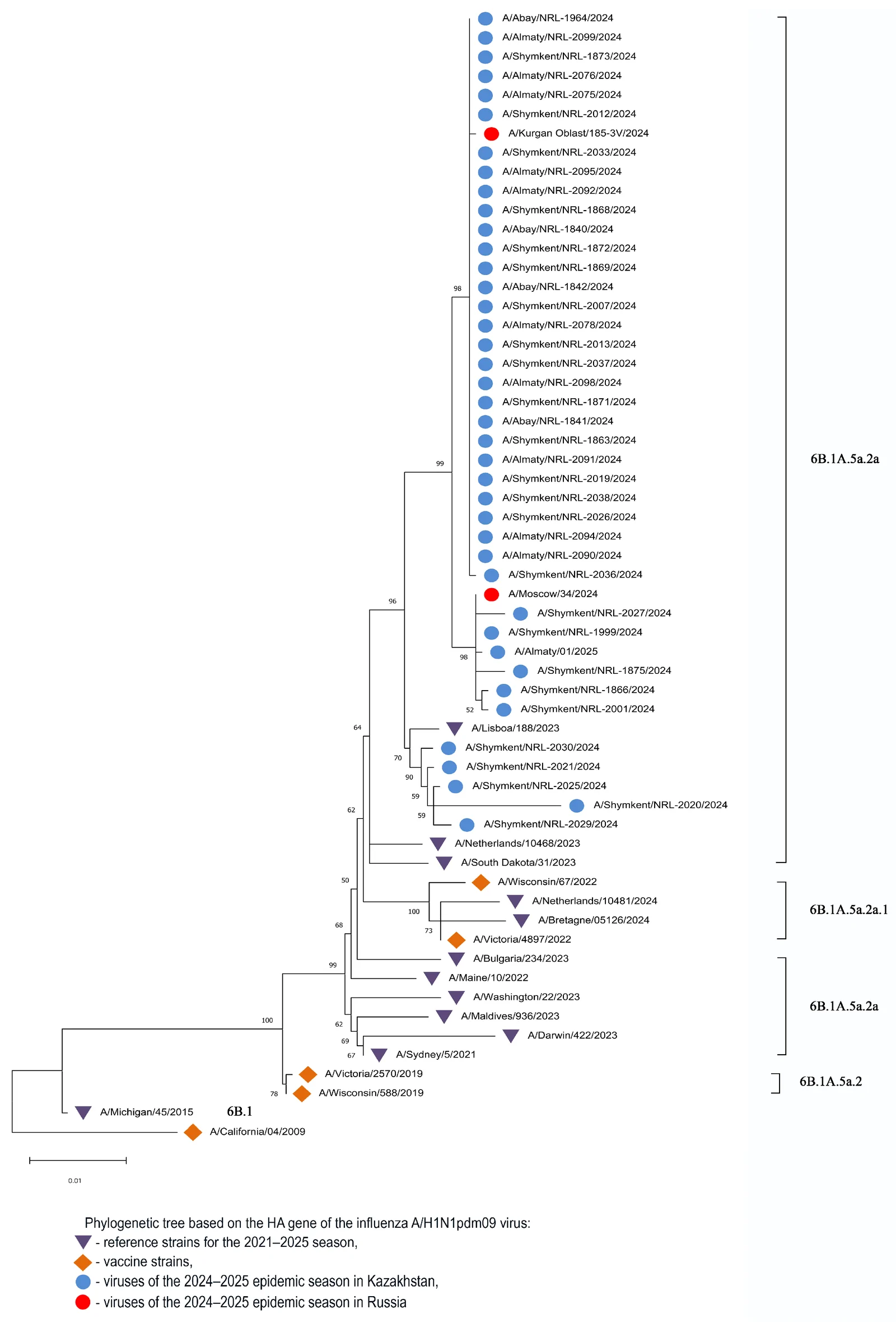
Phylogenetic analysis of nucleotide sequences of the HA gene of influenza viruses A/H1N1pdm09 epidemic season 2024–2025.

**Figure 3:**
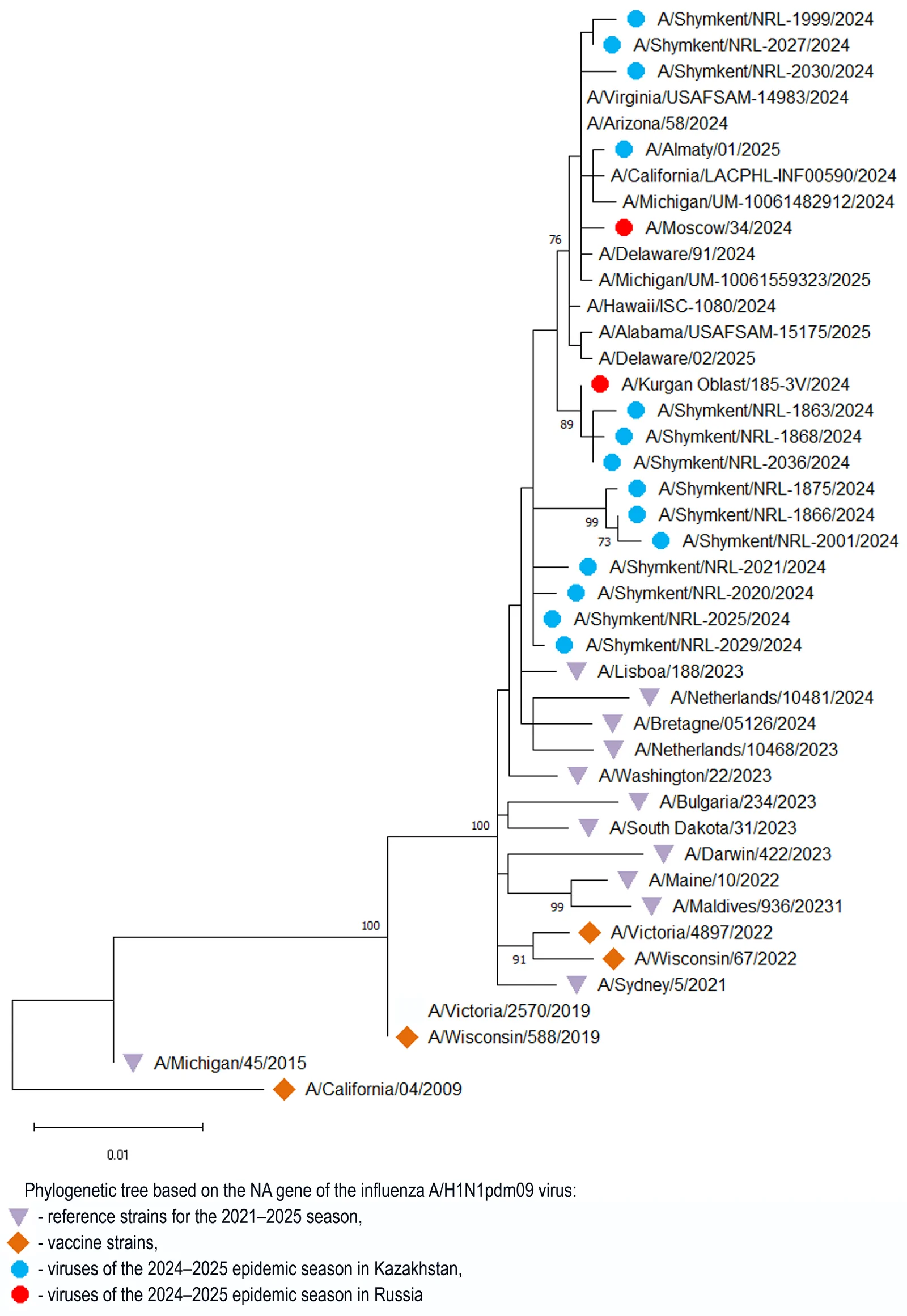
Phylogenetic analysis of nucleotide sequences of the NA gene of influenza viruses A/H1N1pdm09 epidemic season 2024–2025.

To evaluate the genetic diversity and evolutionary trajectories of influenza A(H1N1)pdm09 variants circulating during the 2024–2025 epidemic season in Kazakhstan, a total of 41 isolates were selected for high-throughput sequencing. Influenza A(H1N1)pdm09 isolates were successfully sequenced and assembled for the complete full-length HA gene. However, due to variations in viral load and differential amplifica-tion efficiencies inherent to multiplex next-generation sequencing, complete target se-quence recovery for the NA gene was achieved for of 14 isolates. Consequently, the NA phylogenetic reconstruction was optimized based on these 14 high-quality con-sensus sequences. The respective phylogenetic trees for both surface proteins were recon-structed using the Maximum Likelihood method, incorporating global reference lineages and candidate vaccine viruses recommended by the WHO for the 2024–2025 cycle (Figure 2 and Figure 3).

Phylogenetic analysis of the HA gene allowed to assign the A/H1N1pdm09 strains (A/Shymkent/NRL-2020/2024, A/Shymkent/NRL-2021/2024, A/Shymkent/NRL-2025/2024, A/Shymkent/NRL-2029/2024 and A/Shymkent/NRL-2030/2024) to the C.1.9 genetic clade. These isolates are genetically homogeneous and closely related to the international reference strain A/Lisboa/188/2023 recommended by WHO for the current epidemic season. At the same time, other A/H1N1pdm09 variants circulating in the 2024–2025 season were isolated in Almaty, Shymkent, and the Abay region were assigned to the C.1.9.3 subclade. The vast majority of these C.1.9.3 subclade isolates formed a single, clearly defined genetic cluster with modern strains from Russia (Moscow and the Kurgan region) with high statistical support (bootstrap values of 98–99%).

The tree is constructed by the Maximum Likelihood method using the Tamura model [27]. Branching node values are derived from 1000 bootstrap replicates (bootstrap values ≥60% are indicated at the corresponding nodes) [29]. The symbols indicate: purple triangles—WHO reference strains for the 2024–2025 season; orange diamonds—vaccine strains; blue circles—isolates from Kazakhstan; red circles—isolates from Russia. The scale bar at the bottom corresponds to the number of nucleotide substitutions per site.

In contrast, significant genetic distance was observed between the isolates studied and earlier vaccine strains such as A/Victoria/2570/2019 and A/Wisconsin/588/2019 (clade C), confirming ongoing antigenic drift [32,33]. To assess the evolutionary trajectory, reference strains from intermediate seasons were analyzed, including A/Victoria/4897/2022 (clade D; a strain obtained in chicken embryos) and A/Wisconsin/67/2022 (clade C.1.1; a strain obtained in cell cultures). Although these 2022 vaccine strains belong to the ancestral clade, modern Kazakhstani isolates have separated into a separate subcluster. The reference strain A/Lisboa/188/2023 is a direct evolutionary descendant of this lineage, having accumulated critical mutations within the C.1.9 subclade. Despite this drift, the high degree of genetic relatedness suggests that vaccines based on the 2022 strains may provide partial serological cross-protection, although to achieve maximum prophylactic efficacy, the vaccine formulation needs to be updated in accordance with current WHO recommendations.

Notably, significant genetic divergence was detected in a single A/Shymkent/NRL-2020/2024 strain, which accumulated 19 amino acid mutations compared to the A/Sydney/5/2021 strain. This strain was characterized by the presence of unique, extremely rare substitutions (including C84F, E85K, N101S, E120K, S124K, R130W, F131V, S174T, S221Q, S224N, I231T, R234K, and E252T), indicating that it belongs to a parallel, independent evolutionary branch that is not widespread in the region.

The NA gene phylogenetic tree (Figure 3) showed a stable evolutionary topology reflecting stepwise divergence from the original pandemic variant (A/California/04/2009). The tree consistently splits into the 2015 lineage belonging to clade C (A/Michigan/45/2015), the 2019 cluster (A/Victoria/2570/2019), and the transitional strains of 2021–2022. This is followed by a large global cluster including the 2022–2024 variants from Portugal, the Netherlands, Bulgaria, the Maldives, and the United States (clades C.1.2–C.1.6). At the top of the NA tree, the current 2024–2025 evolutionary lineage is represented predominantly by strains from Kazakhstan. Their close clustering (bootstrap support up to 99%) indicates a localized outbreak caused by a single genetic variant. Notably, the Russian strain A/Kurgan Oblast/185-3V/2024 demonstrates close phylogenetic relationship (89% support) to a specific cluster of isolates from Shymkent (NRL-1863, NRL-1868, and NRL-2036). Strains from early 2025 (e.g., A/Almaty/01/2025 and modern isolates from the United States) occupy the most distant positions at the top of the tree, demonstrating continuous evolution of the NA gene.

### 3.2. Molecular and Mutational Characterization of the Hemagglutinin Gene

The HA is a key protein that enables the virus to enter host cells. It also serves as the primary target for neutralizing antibodies against the influenza virus [34]. The HA molecule of the influenza A(H1N1)pdm09 virus has 5 antigenic sites, which include the following amino acid positions: Sa (121–122 and 150–162), Sb (184–195), Ca1 (163–167, 200–202 and 232–235), Ca2 (133–139 and 218–219), Cb (67–72) [35,36,37].

The primary structure of the HA1 subunit was characterized using the GISAID FluSurver tools [38]. Comparative mutational analysis relative to the reference strain A/Sydney/5/2021(H1N1) revealed that the overwhelming majority of regional isolates from the 2024–2025 season (forming the major C.1.9.3 subclade cluster) exhibited a highly conservative and homogeneous mutational profile defined by a stable signature of nine fixed substitutions: S100P, N111D, T137A, I183V, K186Q, A233T, R240Q, I435V, and I527T. Conversely, a distinct group of basal clade C.1.9 strains (NRL-2021, NRL-2025, NRL-2029, and NRL-2030) lacked the S100P and I183V mutations, thereby retaining the ancestral residues 100S and 183I.

To assess the evolutionary trajectory of the receptor binding site (RBS), the dynamics of loop 220 were analyzed. Although highly virulent mutations such as HA-D222G/N (H1 numbering; corresponds to D225G/N in H3 numbering) were completely absent, an alternative HA-R222K substitution was identified. In the A/Shymkent/NRL-2001/2024 strain, the R222K substitution arose as a single adaptive co-event against the background of the standard regional lineage C.1.9.3. At the same time, in the A/Shymkent/NRL-1875/2024 strain, the R222K substitution was combined with sporadic mutations R221W and V190I located in close proximity to the RBS. The key amino acid substitutions identified in the main structural domains of the HA gene are presented in Table 5.

Against this uniform genetic background, several isolates displayed unique, sporadic amino acid substitutions:

A/Shymkent/NRL-1866/2024: molecularly diverged from the dominant regional profile by retaining the ancestral lysine residue at position 186 (186K), failing to acquire the fixed K186Q substitution shared by the rest of the 2024–2025 cohort.

A/Shymkent/NRL-1875/2024: Displays a rare mutation cascade: 190I, 221W, and 222K.

A/Shymkent/NRL-2001/2024: Harbors an isolated R222K substitution.

A/Shymkent/-2020/2024: Phylogenetically distinct, showing an isolated evolutionary position with 19 amino acid substitutions, including 13 unique mutations: C84F, E85K, N101S, E120K, S124K, R130W, F131V, S174T, S221Q, S224N, I231T, R234K, and E252T.

Mapping the substitutions onto the five known immunodominant antigenic sites of HA1 revealed targeted modifications in key structural regions, supported by auxiliary and framework mutations that maintain the overall stability of the altered protein conformation:

Site Sb (Positions 183 and 190): The fixed V183Q substitution and the sporadic V190I (in strain NRL-1875) alter the α-helix bordering the RBS. Mutations in this site possess high immunodominant potential and are strongly linked to neutralizing antibody escape.

Site Ca1 (Positions 231 and 234): Accommodates fixed A231T and R234Q mutations located at the monomer-monomer junctions of the HA trimer head, indicating a combined rearrangement of the virus’s antigenic landscape.

Site Ca2 (Positions 221 and 222): Features sporadic R221W and R222K substitutions (in strains NRL-1875 and NRL-2001). This region (loop 220) deserves special attention as it ensures the structural stability of the virus’s association with host sialic acid receptors.

Auxiliary and Framework Positions: The fixed substitutions S84P (located near the base of the globular domain), N111D (localized in the obscured region of the stem/lower head), along with S174V and G435T (within the HA2 domain), act as conservative framework mutations that preserve structural integrity amid antigenic drift.

Spatial mapping of the identified amino acid substitutions showed that the majority of fixed mutations in the 2024–2025 season strains affect key immunodominant epitopes on the globular head of HA1, classically divided into five sites [39,40]. In particular, the A231T substitution is localized in the Sa antigenic site, and the V183Q substitution is in the Sb site, located in close proximity to the receptor-binding site (RBS) [41]. The presence of mutations in the Ca1 sites (A231T, R234Q) indicates a combined nature of a structural rearrangement of the antigenic landscape of the virus.

The high genetic relatedness between the studied isolates and current WHO standards suggests preserved serological cross-reactivity, though the observed drift underscores the necessity of the vaccine strain updates [42]. All generated sequences for the Shymkent and Almaty isolates were deposited in the GISAID database (accession numbers are listed in the Supplementary Materials Table S1).

### 3.3. Molecular and Mutational Characterization of the Neuraminidase Gene

Protein FluSurver-based analysis of the NA amino acid sequences revealed high homogeneity among the investigated Kazakhstani strains, with sequence identity ranging from 98.93% to 99.79% (at 99.79% length coverage) compared to the reference seasonal line. Mutational screening relative to the historical reference strain A/Wisconsin/588/2019 identified a total of 15 unique amino acid mutations across the NA gene, providing clear molecular corroboration for the observed phylogenetic topologies.

The overwhelming majority of the Kazakhstani 2024–2025 isolates exhibited a highly consolidated and uniform evolutionary framework defined by a stable signature profile of fixed or high-frequency substitutions, including I46V (*n* = 4), S52N (*n* = 7), V80I, S200N, M264T, and V414M (accession numbers are listed in the Supplementary Materials Tables S2 and S3). Notably, the most prevalent non-synonymous change was the V98A substitution detected across 14 profiled samples, establishing it as an absolute genetic marker for this circulating regional cohort. Based on these background mutational profiles, the analyzed isolates further segregated into two distinct sub-clusters with linked substitutions:

- Cluster 1 [V98A + D199N + M314I]: identified in 3 isolates (A/Shymkent/NRL-1866/2024, A/Shymkent/NRL-1875/2024, and A/Shymkent/NRL-2001/2024).
- Cluster 2 [S52N + V98A + V234I]: identified in 4 isolates (A/Almaty/01/2025, A/Shymkent/NRL-1999/2024, A/Shymkent/NRL-2027/2024, and A/Shymkent/NRL-2030/2024).

Significantly, the NA mutational spectrum accurately mirrored the internal divergence identified in the HA gene. The cluster of isolates corresponding to the proposed parental subclade C.1.9 (NRL-2021, NRL-2025, NRL-2029, and NRL-2030) was molecularly distinguished by the absolute retention of the ancestral 234V residue. Conversely, the remaining regional isolates positioned within the further drifted descendant C.1.9.3 subclade had uniformly transitioned to the 234I variant.

Furthermore, several individual isolates exhibited unique, hyper-sporadic amino acid replacements representing nine single occurrences (A75V, V80M, R220K, I223R, S247N, D324E, G382R, K389T, and G414E). Among these, a localized G324R replacement was unique to the highly divergent A/Shymkent/NRL-2020/2024 variant, while the unique V80M and D314L substitutions were identified exclusively in strain A/Shymkent/NRL-2001/2024.

Crucially, two critical point mutations associated with altered antiviral susceptibility were identified in specific Kazakhstani strains: an unconventional I223R substitution in A/Shymkent/NRL-1999/2024 and a sporadic S247N mutation in A/Shymkent/NRL-2030/2024. Residue 223 is spatially adjacent to the enzyme catalytic pocket, while position 247 constitutes a known drug-susceptibility locus. These specific NA positions require ongoing molecular monitoring, as mutations at these sites are historically documented to reduce viral susceptibility to neuraminidase inhibitors, such as oseltamivir, either independently or when acting synergistically with accompanying framework conversions [43,44,45,46]. The key amino acid substitutions detected across the primary functional and structural domains of the NA gene are summarized in Table 6.

### 3.4. Serological Surveillance and Population Humoral Immunity

**Figure 4:**
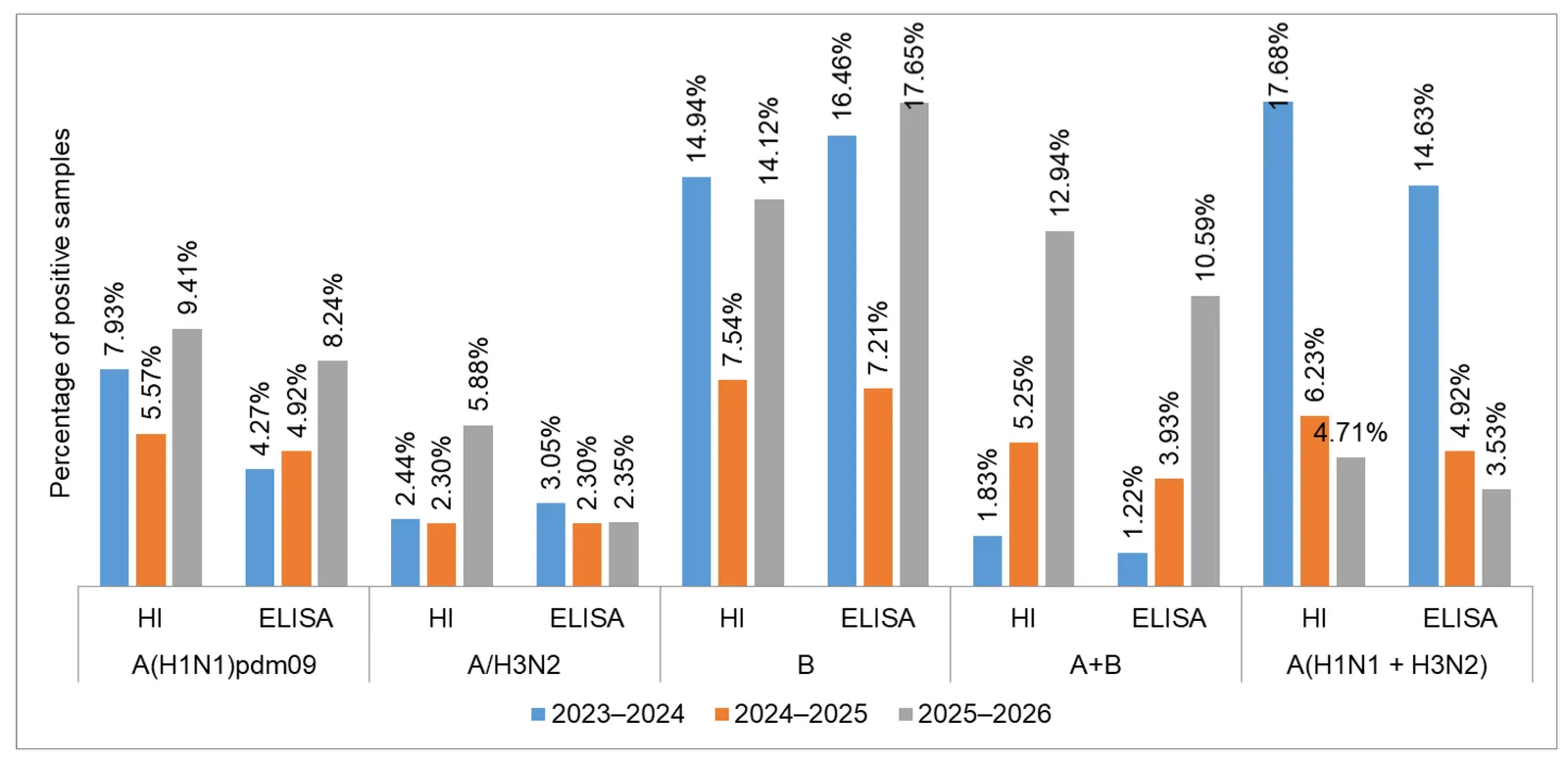
Detection of antibodies against influenza virus strains using HI assay and ELISA during the 2020–2025 epidemic periods.

To identify herd immunity to influenza, serological studies were conducted using the HI and ELISA methods [47]. Serological analysis of 718 serum samples revealed that antibodies to influenza viruses were detected in 47.06% of cases (n = 269) over the entire study period (Figure 4). The highest antibody detection rate was consistently observed for influenza B virus throughout all three seasons (reaching 16.46% and 17.65% in ELISA). In the 2024–2025 season (orange bars), the detection of positive samples decreases, which is especially noticeable for influenza B and mixed group A (H1N1 + H3N2). However, in the A + B category, an increase in antibody rates is observed, where the proportion of positive findings increased to 12.94% (HI) and 10.59% (ELISA). In the 2025–2026 season (gray bars), an increase in seropositivity is also observed for the A/H1N1pdm (increase to 9.41% by HI) and A/H3N2 (increase to 5.88% by HI) viruses.

When comparing serum tests using HI and ELISA, both methods show a high degree of correlation—the graphical trends (rises and declines) for HI and ELISA within each group are virtually synchronous. Minor discrepancies in percentages can be explained by the different sensitivity and specificity of the tests. The HI and ELISA data indicate the circulation of influenza A and B viruses, with a predominance of immunity to influenza B viruses and mixed infections.

## 4. Discussion

During laboratory and epidemiological monitoring of influenza virus circulation in Kazakhstan from 2023 to 2026 reveals a distinct cyclical shift in the etiological structure of seasonal pathogens, aligning with global trends [31,48,49,50,51]. According to the Global Influenza Program (FluNet), the overall detection rate of respiratory viruses in Kazakhstan during this multi-year period was 7.19% (3101/43,109). Within the structure of positive findings, influenza A(H3N2) accounted for 5.92% (2554/43,109), followed by influenza B (Victoria lineage) at 0.69% (2,97/43,109), and influenza A(H1N1)pdm09 at 0.57% (247/43,109).

Age-stratified analysis showed that the overall influenza burden was distributed unevenly across the population (χ^2^ = 8.132; df = 3; *p* = 0.043). The highest overall virus detection rate (positivity) was observed among the economically active population aged 18–65 years (10.30%), which is likely due to high social mobility and professional contacts. However, statistical analysis of individual virus types and subtypes—influenza A (*p* = 0.798), influenza B (*p* = 0.968), A/H1N1pdm09 (*p* = 0.105), and A/H3N2 (*p* = 0.129)—did not reveal any statistically significant age-related differences. For the primary study target—the A/H1N1pdm09 subtype—a slight increase (1.78%) was observed in the adult group, but overall, its prevalence did not show any distinct age-specific patterns. This uniform background circulation facilitated the emergence of sporadic adaptive mutations (e.g., HA-R222K) in various age groups without a clear sociodemographic link.

However, these cumulative figures obscure sharp inter-seasonal transitions. The 2023–2024 and 2025–2026 epidemic seasons were characterized by the near-absolute dominance of the A(H3N2) subtype. For instance, in 2023, out of 13,654 samples tested via sentinel and non-sentinel surveillance, 675 positive cases were verified, with A(H1N1)pdm09 accounting for a negligible 0.59% (4/675). Conversely, the 2024–2025 season witnessed a profound shift in the etiological paradigm: the leading role shifted to the A(H1N1)pdm09 virus, which reached a rate of 1.73% in the overall structure, while A(H3N2) circulation dropped to a minimum of 0.19%. In 2024 alone, out of 11,426 samples analyzed, 563 positive cases were confirmed, with the A(H1N1)pdm09 strain comprising 42.98% (242/563) of the burden.

In terms of demographic impact, real-time RT-qPCR screening revealed that the 2024–2025 H1N1 wave retained its classic epidemiological specificity, heavily involving younger cohorts [52,53]. Children and adolescents under 17 years of age represented the primary vector and infection reservoir, accounting for 60.31% of the total sample (and 58.90% of all PCR-positive cases). The adult population (18–65 years) comprised 28.76%, while the elderly cohort (≥65 years) accounted for 10.93% of the sample (and 10.27% of positive findings). These results correlate with international data confirming that organized pediatric groups drive seasonal epidemics due to high-density social contact networks in educational institutions [54].

On a global scale, the post-pandemic period highlighted the phenomenon of viral interference, where SARS-CoV-2 fundamentally altered modern influenza circulation, causing shifts in traditional seasonality and the complete eradication of the influenza B/Yamagata evolutionary lineage due to stringent quarantine measures and niche competition. Following a period of relative suppression, the 2024–2025 season saw a sharp international resurgence of influenza A(H1N1)pdm09, matching or exceeding severe historical indicators like the 2017–2018 season in the United States. This massive surge in influenza, coupled with the relative suppression of SARS-CoV-2 during the same window, strongly supports the existence of competitive viral interference [55,56,57,58,59].

The post-pandemic evolution of the influenza A(H1N1)pdm09 virus is driven by the steady accumulation of point mutations via antigenic drift [55,60]. Genetic characterization of the Kazakhstani isolates from the 2024–2025 epidemic season demonstrated that these strains belong to the dominant global clade 6B.1A.5a.2a (subclade C.1.9/C.1.9.3) [61]. The identification of a highly fixed, uniform mutational backbone—comprising S100P, N111D, T137A, I183V, K186Q, A233T, R240Q, and I435V—underscores the pronounced genetic homogeneity of the virus circulating within the region. This consolidated molecular profile explains the monophyletic architecture of the reconstructed phylogenetic tree.

From a structural perspective, these fixed substitutions target critical structural and antigenic determinants of the HA molecule. The I183V and K186Q substitutions modify regions directly associated with viral antigenicity, with K186Q representing a recurrent evolutionary marker of seasonal H1N1 selection. Mutations S100P, N111D, and T137A alter specific antigenic determinants and potentially modulate receptor-binding dynamics. Conversely, structural rearrangements like A233T and R240Q appear to optimize protein stability or localized immunogenicity, whereas I435V and I527T function primarily as framework mutations that maintain trimer integrity without altering net antigenicity [62]. Taken together, this fixed mutational complex at the Sa, Sb, and Ca1 sites underpins the observed antigenic drift, driving the evolutionary divergence of modern regional strains from earlier vaccine standards.

A key finding of this study was the identification of individual isolates carrying sporadic mutations, reflecting ongoing microevolution of the virus in situ. Amino acid residues in the region 190–225 (H1 numbering) of the HA1 subunit are spatially adjacent to the cellular receptor binding site (RBS) and play a crucial role in determining the specificity of interaction with host receptors. Although the Kazakhstani sample was completely devoid of highly virulent mutations such as HA-D222G/N (corresponds to D225G/N in H3 numbering), traditionally associated with severe disease, fatal lower respiratory tract infections and tropism for alveolar cells [58], an alternative substitution HA-R222K was found in the A/Shymkent/NRL-1875/2024 and NRL-2001/2024 strains [63]. Unlike the hypervirulent D222G/N variants, the R222K substitution acts as an evolutionary mechanism for fine-tuning host adaptation. The R222K mutation, located in loop 220 (which stabilizes interaction with human α-2,6-sialic acid receptors), alters receptor binding specificity. This mutation likely serves as a tool for gradual virus establishment in the population, optimizing transmission efficiency without a sharp increase in virulence [64]. The combination of R222K with sporadic R221W and V190I mutations (located in or near the RBS), as well as the presence of a unique E252T substitution in the A/Shymkent/NRL-2020/2024 strain, indicate functional plasticity of this region and highlight the need for careful molecular surveillance [65]. Furthermore, the pronounced phylogenetic isolation of the single A/Shymkent/NRL-2020/2024 strain indicates a short-term introduction of a distinct subvariant or the existence of a parallel, independent evolutionary lineage belonging to the ancestral (legacy) clade or proposed subclade (proposed subclade C.1.9), which has not achieved widespread regional dominance compared to the circulating subclade C.1.9.3. Remarkably, the HA-R222K mutation is not unique to any single lineage; it arises periodically in phylogenetically distinct influenza A viruses (e.g., H1N1 and H5N1) during host replication (intrahost microevolution). This recurrence allows us to consider the R222K substitution as a molecular marker of a highly directed, non-random evolutionary process. The conservative nature of this amino acid substitution—preserving the positive charge of the residue with a minor change in the side chain geometry—combined with a minimal genetic barrier (requiring only one nucleotide substitution) and a pronounced selective advantage in mammalian anatomical niches predetermines a natural return of viral populations to this specific evolutionary scenario. As a consequence, this mutation potentially modulates the affinity of the virus for host sialic acid receptors, highlighting the critical importance of continuous long-term monitoring of antigenic drift at position 222 [64,66].

An important aspect of our epidemiological analysis is the relationship between the baseline seropositivity of the population and active virus shedding (detectable nucleotide sequences). It should be clarified that combining data into groups (A + B and H1N1 + H3N2) does not imply the presence of clinically confirmed mixed infections, but serves as an indicator of the overall circulation of the virus in the region. When identifying cohorts with monoinfection, a clear nonlinear correlation was found between seropositivity and active virus shedding. High virus detection in the acute phase did not always correspond to elevated titers of neutralizing or binding antibodies in the same patient; this phenomenon is usually explained by the delay (lag) in the primary immune response. Furthermore, interpretation of serological profiles requires consideration of participant vaccination data. In Kazakhstan, in the 2024–2025 season, routine seasonal vaccination is the main factor in the formation of preexisting cross-reactive antibodies. Although precise data on individual vaccination history were missing for a portion of our cohort—which is a confounding factor when distinguishing post-vaccination titers from the immune response to natural infection—the high baseline seropositivity to reference strains indicates significant immunological imprinting due to both previous exposure to the pathogen and vaccination campaigns conducted in the region.

Analysis of the NA gene demonstrated evolutionary continuity, tracing structural branching from the ancestral pandemic root (A/California/04/2009) to contemporary seasonal lineages. The V98A mutation was identified as a fixed phylogenetic marker across 100% of the investigated regional samples. This finding aligns with global surveillance data, which reports the rapid expansion of V98A to a frequency of 63.74% worldwide following its initial detection in early 2025. Similarly, the S52N mutation, present in seven Kazakhstani isolates, mirrors a broader international trend (21.21% global prevalence). Structurally, S52N abrogation of a potential N-glycosylation site at position 50 may alter the antigenic properties or structural dynamics of the NA head.

The segregation of local isolates into two recurring genetic sub-clusters—[V98A + D199N + M314I] and [S52N + V98A + V234I]—suggests a multi-lineage circulation layout. Concurrently, other amino acid substitutions (D324E, I46V, V80M, G414E, M314I, G382R, and K389T) were mapped outside the catalytic domain. These substitutions represent well-documented adaptive or compensatory mutations common in modern H1N1pdm09 lineages, serving to stabilize NA enzyme conformation amidst ongoing drift.

The identification of specific NA mutations linked to reduced antiviral susceptibility represents a critical epidemiological finding [43,44,45,46]. The A/Shymkent/NRL-1999/2024 sample carried the I223R substitution, a well-known marker capable of increasing the half-maximal inhibitory concentration (IC_50_) of oseltamivir by 11- to 28-fold, thereby compromising standard Tamiflu regimens [44,45,46]. Additionally, the S247N mutation found in strain A/Shymkent/NRL-2030/2024 is a recognized viral escape marker that mediates moderately reduced susceptibility to oseltamivir while maintaining robust human-to-human transmissibility [43,44,45,46].

The primary clinical threat associated with positions 223 and 247 is their potential synergistic interaction with the classic H275Y resistance mutation. The emergence of multi-mutant variants combining H275Y with I223V/R or S247N can trigger an extreme, >1000-fold surge in oseltamivir resistance, completely neutralizing the drug’s therapeutic efficacy [43,44,45,46,60]. Rule-based in silico susceptibility screening of the investigated Kazakhstani isolates revealed a highly consolidated sensitivity framework, with one exception. Out of the analyzed cohort, the overwhelming majority of the strains were genotypically predicted to maintain full susceptibility to neuraminidase inhibitors (oseltamivir, zanamivir, peramivir, and laninamivir). Furthermore, the strain A/Shymkent/NRL-2030/2024, which harbored the sporadic NA-S247N substitution, was classified as sensitive to all tested therapeutic agents, indicating that this minor mutation was an isolated event within the circulating regional genetic background and did not drive predictions of functional drug resistance (accession numbers are listed in the Supplementary Materials Table S4) [67].

Crucially, the automated in silico annotation pipeline uncovered a distinct predicted resistant profile in the solitary strain A/Shymkent/NRL-1999/2024. This specific isolate was marked as resistant to oseltamivir, peramivir, and zanamivir, while retaining predicted susceptibility exclusively to laninamivir. On a molecular level, this predicted multidrug-resistance profile highlights the potential disruptive impact of the NA-I223R mutation identified adjacent to the enzyme&apos;s catalytic center, where structural alterations (V116, I117, E119, Q136, D199, I223) are known to influence inhibitor binding. Remarkably, this functional resistance profile was predicted to develop independently, in the absolute absence of the canonical NA-H275Y anchor mutation. This genotypic prediction suggests that certain non-synonymous remodeling steps within the neuraminidase active site may be sufficient to independently alter drug-binding dynamics, warranting intensive molecular surveillance.

Furthermore, the D199N substitution—detected in three Shymkent isolates—is situated adjacent to the catalytic center, a region where structural alterations (V116, I117, E119, Q136, D199, and I223) are known to influence inhibitor binding. Crucially, while the majority of isolates carrying baseline configurations are predicted to remain sensitive, in silico screening suggests that the accumulation of specific active-site variations, such as the NA-I223R mutation in strain A/Shymkent/NRL-1999/2024, is sufficient to independently disrupt this binding pocket and drive multi-drug resistance predictions even in the absence of canonical anchor mutations [68,69]. 

Certain limitations of this study should be noted. First, the assessment of antigenic similarity between the Kazakhstani isolates and current WHO vaccine strains was based solely on genotypic sequence alignment results, rather than on empirical phenotypic methods such as the HI test. Second, phenotypic validation of viral susceptibility to neuraminidase inhibitors (oseltamivir and zanamivir) using an in vitro enzymatic assay to determine the experimental IC50 was not performed in this study. The antiviral resistance characteristics described herein rely strictly on in silico screening via the GISAID/FluSurver reference monitoring pipelines, which match identified mutations against databases of known resistance markers. Although these genotypic data strongly suggest the emergence of an independent multidrug-resistance marker in the A/Shymkent/NRL-1999/2024 strain, future regional surveillance frameworks must include comprehensive, empirical phenotypic and serological assays to dynamically validate these molecular evolutionary trends.

This study analyzes the genetic characteristics and circulating patterns of influenza A/H1N1pdm09 viruses in Kazakhstan and border regions of Russia during the 2024–2025 season, highlighting active trans-Eurasian exchange and high antigenic identity with current vaccine strains [32]. Findings underscore the critical importance of continuous genomic surveillance to monitor viral evolution and guide public health interventions against seasonal influenza [60,70,71,72]. Because these regional isolates track tightly with the global reference strain A/Lisboa/188/2023, they confirm high antigenic identity with the current WHO-recommended vaccine formulation. This structural alignment confirms the high protective potential and clinical relevance of specific prophylaxis implemented during the 2024–2025 epidemic season.

## 5. Conclusions

Thus, based on molecular and epidemiological surveillance of influenza virus circulation in Kazakhstan from 2023 to 2026 revealed a pronounced cyclical pattern in the pathogen structure, where the absolute dominance of the influenza A(H3N2) subtype during the 2023–2024 and 2025–2026 seasons was interrupted by a significant epidemiological resurgence of the A(H1N1)pdm09 subtype during the 2024–2025 season.

Circulating 2024–2025 A(H1N1)pdm09 strains demonstrated continuous molecular evolution and high genetic homogeneity, tracking entirely within global clade 6B.1A.5a.2a (subclade C.1.9/ C.1.9.3). The fixation of a specific complex of amino acid substitutions across key antigenic sites (Sa, Sb, Ca1) underscores a systematic antigenic drift that drives the evolutionary divergence of modern regional strains away from ancestral vaccine standards.

Concurrently, the identification of solitary isolates harboring sporadic substitutions within the HA 190–225 region (specifically HA-R222K and 252K) reflects active, localized viral microevolution in situ, pointing toward ongoing structural tuning for optimized host adaptation. Furthermore, genomic screening successfully flagged rare regional variants carrying critical NA mutations (I223R and S247N) associated with reduced susceptibility to NA inhibitors.

Although rule-based in silico screening predicts that most of the investigated Kazakhstani strains retain baseline genomic susceptibility to oseltamivir and zanamivir, the identification of the predicted multidrug-resistant A/Shymkent/NRL-1999/2024 strain highlights a tangible risk of the natural selection and dissemination of resistant lineages. Ultimately, the tight phylogenetic clustering of Kazakhstani and cross-border Russian isolates with the reference strain A/Lisboa/188/2023 confirms excellent antigenic match with the updated WHO-recommended vaccine formulation, validating the relevance of specific prophylaxis and highlighting the critical need for expanded, continuous genomic monitoring of respiratory pathogens across the trans-Eurasian region.

## Figures and Tables

**Table 1:** Geographic distribution and specimen types of clinical samples collected during the 2023–2026 epidemic seasons.

Geographic Region/Locality	Nasopharyngeal Swabs, *n* (%)	Blood Serum, *n* (%)	Total Samples, *n* (%)
Southern Kazakhstan			
Almaty	362 (20.62)	249 (34.68)	611 (24.70)
Zhambyl Region	62 (3.53)	—	62 (2.51)
Turkestan Region	125 (7.12)	—	125 (5.05)
Kyzylorda Region	65 (3.70)	—	65 (2.63)
Subtotal	614 (34.97)	249 (34.68)	863 (34.88)
Northern Kazakhstan			
North Kazakhstan Region	349 (19.87)	200 (27.86)	549 (22.19)
Kostanay Region	114 (6.49)	—	114 (4.61)
Pavlodar Region	44 (2.51)	—	44 (1.78)
Subtotal	507 (28.87)	200 (27.86)	707 (28.58)
Western Kazakhstan			
West Kazakhstan Region (Uralsk)	39 (2.22)	—	39 (1.58)
Mangystau Region	28 (1.59)	—	28 (1.13)
Subtotal	67 (3.82)	—	67 (2.71)
Eastern Kazakhstan			
East Kazakhstan Region	214 (12.19)	130 (18.11)	244 (9.86)
Abay Region	73 (4.16)	—	73 (2.95)
Subtotal *	287 (16.34)	130 (18.11)	417 (16.86)
Central Kazakhstan			
Karaganda Region	281 (16.00)	139 (19.36)	420 (16.98)
Subtotal	281 (16.00)	139 (19.36)	420 (16.98)
Total	1756 (100.0)	718 (100.0)	2474 (100.0)

* Note: “—“ indicates no sample collection. In Eastern Kazakhstan sub-localities, ensure the individual values sum up to the subtotal of 287 nasopharyngeal swabs, as 54 samples are internally unaccounted for in the sub-item breakdown.

**Table 2:** Oligonucleotide primer and probe sequences used for the detection and subtyping of influenza viruses by RT-qPCR.

Type/Subtype	Target Gene	Oligonucleotide Name	Sequence (5′ → 3′)
Influenza A virus	*M*	FLUAM-7-F	CTTCTAACCGAGGTCGAAACGTA
		FLUAM-161-R	GGTGACAGGATTGGTCTTGTCTTTA
		FLUAM-49-P	TCAGGCCCCCTCAAAGCCGAG
Influenza B virus	*HA*	FLUBHA-940-F	AAATACGGTGGATTAAACAAAAGCAA
		FLUBHA-1109-R	CCAGCAATAGCTCCGAAGAAA
		FLUBHA-994-P	CACCCATATTGGGCAATTTCCTATGGC
Influenza A(H1N1)pdm09	*HA*	H1pdm-169-F	AAACTATGCAAACTAAGAGGGGT
		H1pdm-297-R	TGTTTCCACAATGTAGGACCA
		H1pdm-244-P	CCAGAGTGTGAATCACTCTCCACA
A(H3)	*HA*	H3-266-F	ACCCTCAGTGTGatGGCTTTCAAA
		H3-373-R	TAAGGGAGGCATAATCCGGCACAT
		H3-315-P	ACGAAGCAAAGCCTACAGCAACTGTT
Internal control	RP (Human RNase P)	RP-F	AGATTTGGACCTGCGAGCG
		RP-R	GAGCGGCTGTCTCCACAAGT
		RP-P	TTCTGACCTTAAGAAGGCTCTGCGCG

Table note: Degenerate nucleotides are designated according to the IUPAC nomenclature (R, Y, B, D, V). The commercial quencher modification (/TAO/) was omitted from the RP-P probe sequence to ensure table uniformity.

**Table 3:** Screening of nasopharyngeal samples collected in Kazakhstan in 2023–2025 using RT-qPCR for Influenza viruses.

Virus Type	2023–2024 (Y1) *n* = 465	2024–2025 (Y2) *n* = 521	2025–2026 (Y3) *n* = 770	Total *N* = 1756	Overall *p*-Value	Pairwise Padj (Holm Method)
Influenza A nonsubtyped	26/5.59% (3.68–8.11%)	5/0.96% (0.31–2.22%)	13/1.69% (0.90–2.87%)	44/2.51% (1.83–3.35%)	<0.001	Y1 vs. Y2: <0.001 Y2 vs. Y3: 0.338 Y1 vs. Y3: <0.001
Influenza B	11/2.37% (1.19–4.20%)	9/1.73% (0.79–3.25%)	1/0.13% (0.00–0.72%)	21/1.20% (0.74–1.83%)	<0.001	Y1 vs. Y2: 0.505 Y2 vs. Y3: 0.003 Y1 vs. Y3: <0.001
A(H1N1)pdm09	1/0.22% (0.01–1.19%)	14/2.69% (1.48–4.47%)	1/0.13% (0.00–0.72%)	16/0.91% (0.52–1.48%)	<0.001	Y1 vs. Y2:0.002 Y2 vs. Y3: <0.001 Y1 vs. Y3: 1.000
A(H3N2)	41/8.82% (6.42–11.74%)	1/0.19% (0.01–1.07%)	23/2.99% (1.91–4.44%)	65/3.70% (2.87–4.69%)	<0.001	Y1 vs. Y2: <0.001 Y2 vs. Y3: <0.001 Y1 vs. Y3: <0.001
Influenza in total	79/16.99% (13.68–20.74%)	29/5.57% (3.76–7.91%)	38/4.94% (3.52–6.72%)	146/8.31% (7.06–9.72%)	<0.001	Y1 vs. Y2: <0.001 Y2 vs. Y3: 0.612 Y1 vs. Y3: <0.001

Table note: Data are presented as absolute counts/percentages (95% confidence intervals). Italics are used to designate statistical parameters, sample size variables (*n*, *N*, *p*).

**Table 4:** Age-stratified breakdown, positivity rates, and Chi-square independence testing of influenza virus types and subtyped lineages during the 2024–2025 epidemic season in Kazakhstan (*N* = 1756).

Age Group (Years)	Total Specimens (*n*)	Influenza A (*n*, %)	Influenza B (*n*, %)	Subtyped A/H1N1pdm09 (*n*, %)	Subtyped A/H3N2 (*n*, %)	Total Influenza Positive (*n*, %)
**0–4**	416	12 (2.88%)	5 (1.20%)	3 (0.72%)	18 (4.33%)	38 (9.13%)
**5–17**	643	13 (2.02%)	7 (1.09%)	3 (0.47%)	15 (2.33%)	38 (5.91%)
**18–65**	505	14 (2.77%)	7 (1.39%)	9 (1.78%)	22 (4.36%)	52 (10.30%)
**≥65**	192	5 (2.60%)	2 (1.04%)	1 (0.52%)	10 (5.21%)	18 (9.38%)
**Total**	**1756**	**44 (2.51%)**	**21 (1.20%)**	**16 (0.91%)**	**65 (3.70%)**	**146 (8.31%)**
Statistical Analysis	—	*χ*^2^ = 1.015 *df* = 3 *p* = 0.798	*χ*^2^ = 0.256 *df* = 3 *p* = 0.968	*χ*^2^ = 6.142 *df* = 3 *p* = 0.105	*χ*^2^ = 5.666 *df* = 3 *p* = 0.129	*χ*^2^ = 8.132 *df* = 3 ***p* = 0.043**

Table note: Individual percentages within the cells indicate the direct positivity rate calculated relative to the total number of tested specimens within that specific age row. Bold *p*-values indicate statistically significant differences (*p* < 0.05) across the stratified age groups, determined by Pearson’s chi-square test (*df* = 3). Italics are explicitly used to denote statistical parameters and variables (*n*, χ^2^, df, *p*).

**Table 5:** Summary of amino acid substitutions in key functional domains of the HA protein among Kazakhstani A/H1N1pdm09 isolates.

Functional Domain/Region	Amino Acid Position	Reference Residue (A/Sydney/5/2021)	Identified Substitution(s)	Key Isolates Involved	Functional/Evolutionary Significance
Receptor-Binding Site (RBS)/Loop 220	222 (H1 num.) (225 in H3)	R (Arg)	K (Lys)	NRL-1875/2024 NRL-2001/2024	Fine-tuning host adaptation; modulates affinity to human α-2,6 sialic receptors; baseline antigenic drift.
Receptor-Binding Site (RBS)	190	V (Val)	I (Ile)	NRL-1875/2024	Located within the RBS; spatial coordination of receptor specificity.
Loop 220 Adjacent Region	221	S (Ser)	W (Trp) Q (Gln)	NRL-1875/2024 NRL-2020/2024	Sporadic microevolution in situ; functional plasticity adjacent to loop 220.
Antigenic Sites/Epitopes	100	S (Ser)	P (Pro)	Overwhelming majority (Except C.1.9 cluster)	Fixed signature mutation defining the regional dominant subclade C.1.9.3.
Hyper-Virulent Markers	222 (H1 num.)	D (Asp)	None (D retained)	All isolates	Absent (D222G/N entirely absent); rules out alveolar tropism and severe lower respiratory fatal phenotypes.
Hyper-Divergent Cluster	Multiple (84, 85, 120, 124, 130, 252, etc.)	Varied	C84F, E85K, E120K, S124K, R130W, E252T	NRL-2020/2024	Solitary independent evolutionary branch displaying extreme genetic distance (19 mutations).

Table note: H1 num., H1 numbering system; H3, influenza A(H3N2) virus subtype; RBS, receptor-binding site. All data, amino acid residues, and mutational positions are presented in standard upright roman font.

**Table 6:** Summary of amino acid substitutions in key functional and structural domains of the NA protein relative to the A/Wisconsin/588/2019 reference.

Functional Domain/Structural Region	Amino Acid Position	Reference Residue (A/Wisconsin/588/2019)	Identified Substitution(s)	Key Isolates Involved	Functional/Evolutionary Significance
Active Center/Framework Neighbor	223	I (Ile)	R (Arg)	NRL-1999/2024	Unconventional substitution spatially adjacent to the enzyme catalytic pocket; requires drug-susceptibility screening.
Drug Susceptibility Locus	247	S (Ser)	N (Asn)	NRL-2030/2024	Sporadic mutation; historically monitored for minor impacts on neuraminidase inhibitor binding.
Phylogenetic Subclade Marker	234	V (Val)	I (Ile)	Overwhelming majority (Except C.1.9 cluster)	Clear molecular indicator of internal drift; retention of 234V marks the basal C.1.9 cluster.
Primary Resistance Marker	275	H (His)	None (H retained)	All isolates	Absent (H275Y absent); predicts preserved baseline susceptibility to oseltamivir and zanamivir.
Consolidated Regional Lineage Profile	Multiple (46, 52, 80, 200, 264, 414)	Varied	I46V, S52N, V80I, S200N, M264T, V414M	Overwhelming majority	Monolithic genetic background confirming a uniform regional outbreak of the 2024–2025 season.

## Data Availability

The consensus sequences of the viruses analyzed in this study were submitted to the GISAID EpiFlu™ under the accession numbers reported in the Table 3.

## Supplementary Materials

The following supporting information can be downloaded at: https://www.mdpi.com/article/10.3390/v18101087/s1, Table S1: Mutations in the HA gene relative to the reference strain HA A/Sydney/5/2021(H1N1); Table S2: Mutations in the amino acid sequence of the NA gene of Kazakhstani strains relative to the most suitable reference A/Missouri/11/2025(H1N1); Table S3: Substitutions in the hemagglutinin gene in Kazakhstani strains relative to the reference influenza virus A/Sydney/5/2021(H1N1); Table S4: Strains Sensetivity.

## Abbreviations

The following abbreviations are used in this manuscript:

ARVI	Acute Respiratory Virus Infection
IAV	Influenza A virus
HA	Hemagglutinin
NA	Neuraminidase
RDE	Receptor-Destroying Enzyme
RT-qPCR	Real-time polymerase chain reaction
ELISA	Enzyme-linked immunosorbent assay
HI	Hemagglutinating activity inhibition
WHO	World Health Organization
