# Supplementary material for: Study of Molecular Genetic Characteristics of Influenza A(H1N1) Viruses Identified During Monitoring in Kazakhstan (2023–2026)
Source: Viruses. 2026 Oct 1;18(10):1087. doi: 10.3390/v18101087 (PMC13709524; doi:10.3390/v18101087)
Supplement: Supplementary file 1 [file viruses-18-01087-s001.zip › S4_Strains_Sensetivity.pdf]

Table S4. Strains Sensetivity.

Query	Protein	subtype	drugname	sensitivity	degree of inhibition
EPI3880217|NA|A/Shymkent/NRL-1868	NA	H1N1pdm	oseltamivir	sensitive	
EPI3880217|NA|A/Shymkent/NRL-1868	NA	H1N1pdm	peramivir	sensitive	
EPI3880217|NA|A/Shymkent/NRL-1868	NA	H1N1pdm	laninamivir	sensitive	
EPI3880217|NA|A/Shymkent/NRL-1868	NA	H1N1pdm	zanamivir	sensitive	
EPI3881148|NA|A/Shymkent/NRL-2036	NA	H1N1pdm	oseltamivir	sensitive	
EPI3881148|NA|A/Shymkent/NRL-2036	NA	H1N1pdm	laninamivir	sensitive	
EPI3881148|NA|A/Shymkent/NRL-2036	NA	H1N1pdm	peramivir	sensitive	
EPI3881148|NA|A/Shymkent/NRL-2036	NA	H1N1pdm	zanamivir	sensitive	
EPI3880904|NA|A/Shymkent/NRL-2001	NA	H1N1pdm	zanamivir	sensitive	
EPI3880904|NA|A/Shymkent/NRL-2001	NA	H1N1pdm	peramivir	sensitive	
EPI3880904|NA|A/Shymkent/NRL-2001	NA	H1N1pdm	laninamivir	sensitive	
EPI3880904|NA|A/Shymkent/NRL-2001	NA	H1N1pdm	oseltamivir	sensitive	
EPI3880429|NA|A/Shymkent/NRL-1875	NA	H1N1pdm	zanamivir	sensitive	
EPI3880429|NA|A/Shymkent/NRL-1875	NA	H1N1pdm	laninamivir	sensitive	
EPI3880429|NA|A/Shymkent/NRL-1875	NA	H1N1pdm	peramivir	sensitive	
EPI3880429|NA|A/Shymkent/NRL-1875	NA	H1N1pdm	oseltamivir	sensitive	
EPI3881009|NA|A/Shymkent/NRL-2020	NA	H1N1pdm	laninamivir	sensitive	
EPI3881009|NA|A/Shymkent/NRL-2020	NA	H1N1pdm	peramivir	sensitive	
EPI3881009|NA|A/Shymkent/NRL-2020	NA	H1N1pdm	zanamivir	sensitive	
EPI3881009|NA|A/Shymkent/NRL-2020	NA	H1N1pdm	oseltamivir	sensitive	
EPI3880902|NA|A/Shymkent/NRL-1999	NA	H1N1pdm	zanamivir	resistant	NI/RI
EPI3880902|NA|A/Shymkent/NRL-1999	NA	H1N1pdm	peramivir	resistant	NI/RI
EPI3880902|NA|A/Shymkent/NRL-1999	NA	H1N1pdm	laninamivir	sensitive	NI
EPI3880902|NA|A/Shymkent/NRL-1999	NA	H1N1pdm	oseltamivir	resistant	RI
EPI3881021|NA|A/Shymkent/NRL-2025	NA	H1N1pdm	oseltamivir	sensitive	
EPI3881021|NA|A/Shymkent/NRL-2025	NA	H1N1pdm	zanamivir	sensitive	
EPI3881021|NA|A/Shymkent/NRL-2025	NA	H1N1pdm	peramivir	sensitive	
EPI3881021|NA|A/Shymkent/NRL-2025	NA	H1N1pdm	laninamivir	sensitive	
EPI3881146|NA|A/Shymkent/NRL-2030	NA	H1N1pdm	oseltamivir	sensitive	NI
EPI3881146|NA|A/Shymkent/NRL-2030	NA	H1N1pdm	peramivir	sensitive	NI
EPI3881146|NA|A/Shymkent/NRL-2030	NA	H1N1pdm	laninamivir	sensitive	NI
EPI3881146|NA|A/Shymkent/NRL-2030	NA	H1N1pdm	zanamivir	sensitive	NI
EPI3881011|NA|A/Shymkent/NRL-2021	NA	H1N1pdm	peramivir	sensitive	
EPI3881011|NA|A/Shymkent/NRL-2021	NA	H1N1pdm	laninamivir	sensitive	
EPI3881011|NA|A/Shymkent/NRL-2021	NA	H1N1pdm	zanamivir	sensitive	
EPI3881011|NA|A/Shymkent/NRL-2021	NA	H1N1pdm	oseltamivir	sensitive	
EPI3881023|NA|A/Shymkent/NRL-2027	NA	H1N1pdm	oseltamivir	sensitive	
EPI3881023|NA|A/Shymkent/NRL-2027	NA	H1N1pdm	laninamivir	sensitive	
EPI3881023|NA|A/Shymkent/NRL-2027	NA	H1N1pdm	peramivir	sensitive	
EPI3881023|NA|A/Shymkent/NRL-2027	NA	H1N1pdm	zanamivir	sensitive	
EPI3879690|NA|A/Shymkent/NRL-1863	NA	H1N1pdm	oseltamivir	sensitive	
EPI3879690|NA|A/Shymkent/NRL-1863	NA	H1N1pdm	peramivir	sensitive	
EPI3879690|NA|A/Shymkent/NRL-1863	NA	H1N1pdm	laninamivir	sensitive	
EPI3879690|NA|A/Shymkent/NRL-1863	NA	H1N1pdm	zanamivir	sensitive	
EPI3881092|NA|A/Shymkent/NRL-2029	NA	H1N1pdm	oseltamivir	sensitive	
EPI3881092|NA|A/Shymkent/NRL-2029	NA	H1N1pdm	zanamivir	sensitive	
EPI3881092|NA|A/Shymkent/NRL-2029	NA	H1N1pdm	peramivir	sensitive	

EPI3881092|NA|A/Shymkent/NRL-2029	NA	H1N1pdm	laninamivir	sensitive	
EPI3879956|NA|A/Shymkent/NRL-1866	NA	H1N1pdm	zanamivir	sensitive	
EPI3879956|NA|A/Shymkent/NRL-1866	NA	H1N1pdm	laninamivir	sensitive	
EPI3879956|NA|A/Shymkent/NRL-1866	NA	H1N1pdm	peramivir	sensitive	
EPI3879956|NA|A/Shymkent/NRL-1866	NA	H1N1pdm	oseltamivir	sensitive	
