# Supplementary material for: Study of Molecular Genetic Characteristics of Influenza A(H1N1) Viruses Identified During Monitoring in Kazakhstan (2023–2026)
Source: Viruses. 2026 Oct 1;18(10):1087. doi: 10.3390/v18101087 (PMC13709524; doi:10.3390/v18101087)
Supplement: Supplementary file 1 [file viruses-18-01087-s001.zip › S1_HA_List_mutations_FluSurver.pdf]

Table S1. Mutations in the HA gene relative to the reference strain HA A/Sydney/5/2021(H1N1)

Query	Clade	Best reference hit	% AA identity	% length coverage	# mutations	List of mutations
A/Abay/NRL-1840/2024	-	HA A/Sydney/5/2021(H1N1)	98.361	96.996	9	**S100P**, **N111D**, **T137A**, **I183V**, **K186Q**, **A233T**, **R240Q**, **I435V**, **I527T** show in structure
A/Abay/NRL-1841/2024	-	HA A/Sydney/5/2021(H1N1)	98.361	96.996	9	**S100P**, **N111D**, **T137A**, **I183V**, **K186Q**, **A233T**, **R240Q**, **I435V**, **I527T** show in structure
A/Abay/NRL-1842/2024	-	HA A/Sydney/5/2021(H1N1)	98.361	96.996	9	**S100P**, **N111D**, **T137A**, **I183V**, **K186Q**, **A233T**, **R240Q**, **I435V**, **I527T** show in structure
A/Abay/NRL-1964/2024	-	HA A/Sydney/5/2021(H1N1)	98.361	96.996	9	**S100P**, **N111D**, **T137A**, **I183V**, **K186Q**, **A233T**, **R240Q**, **I435V**, **I527T** show in structure
A/Almaty/01/2025	-	HA A/Sydney/5/2021(H1N1)	98.543	96.996	8	**S100P**, **N111D**, **T137A**, **K186Q**, **A233T**, **R240Q**, **I435V**, **I527T** show in structure
A/Almaty/NRL-2075/2024	-	HA A/Sydney/5/2021(H1N1)	98.361	96.996	9	**S100P**, **N111D**, **T137A**, **I183V**, **K186Q**, **A233T**, **R240Q**, **I435V**, **I527T** show in structure
A/Almaty/NRL-2076/2024	-	HA A/Sydney/5/2021(H1N1)	98.361	96.996	9	**S100P**, **N111D**, **T137A**, **I183V**, **K186Q**, **A233T**, **R240Q**, **I435V**, **I527T** show in structure

A/Almaty/NRL-2078/2024	-	HA A/Sydney/5/2021(H1N1)	98.361	96.996	9	**S100P**, **N111D**, **T137A**, **I183V**, **K186Q**, **A233T**, **R240Q**, **I435V**, **I527T** show in structure
A/Almaty/NRL-2090/2024	-	HA A/Sydney/5/2021(H1N1)	98.361	96.996	9	**S100P**, **N111D**, **T137A**, **I183V**, **K186Q**, **A233T**, **R240Q**, **I435V**, **I527T** show in structure
A/Almaty/NRL-2091/2024	-	HA A/Sydney/5/2021(H1N1)	98.361	96.996	9	**S100P**, **N111D**, **T137A**, **I183V**, **K186Q**, **A233T**, **R240Q**, **I435V**, **I527T** show in structure
A/Almaty/NRL-2092/2024	-	HA A/Sydney/5/2021(H1N1)	98.361	96.996	9	**S100P**, **N111D**, **T137A**, **I183V**, **K186Q**, **A233T**, **R240Q**, **I435V**, **I527T** show in structure
A/Almaty/NRL-2094/2024	-	HA A/Sydney/5/2021(H1N1)	98.361	96.996	9	**S100P**, **N111D**, **T137A**, **I183V**, **K186Q**, **A233T**, **R240Q**, **I435V**, **I527T** show in structure
A/Almaty/NRL-2095/2024	-	HA A/Sydney/5/2021(H1N1)	98.361	96.996	9	**S100P**, **N111D**, **T137A**, **I183V**, **K186Q**, **A233T**, **R240Q**, **I435V**, **I527T** show in structure
A/Almaty/NRL-2098/2024	-	HA A/Sydney/5/2021(H1N1)	98.361	96.996	9	**S100P**, **N111D**, **T137A**, **I183V**, **K186Q**, **A233T**, **R240Q**, **I435V**, **I527T** show in structure
A/Almaty/NRL-2099/2024	-	HA A/Sydney/5/2021(H1N1)	98.361	96.996	9	**S100P**, **N111D**, **T137A**, **I183V**, **K186Q**, **A233T**, **R240Q**, **I435V**, **I527T** show in structure

A/Shymkent/NRL-1863/2024	-	HA A/Sydney/5/2021(H1N1)	98.361	96.996	9	**S100P**, **N111D**, **T137A**, **I183V**, **K186Q**, **A233T**, **R240Q**, **I435V**, **I527T** show in structure
A/Shymkent/NRL-1866/2024	-	HA A/Sydney/5/2021(H1N1)	98.361	96.996	9	**S100P**, **N111D**, **T137A**, **K186Q**, **A233T**, **R240Q**, **E252K**, **I435V**, **I527T** show in structure
A/Shymkent/NRL-1868/2024	-	HA A/Sydney/5/2021(H1N1)	98.361	96.996	9	**S100P**, **N111D**, **T137A**, **I183V**, **K186Q**, **A233T**, **R240Q**, **I435V**, **I527T** show in structure
A/Shymkent/NRL-1869/2024	-	HA A/Sydney/5/2021(H1N1)	98.361	96.996	9	**S100P**, **N111D**, **T137A**, **I183V**, **K186Q**, **A233T**, **R240Q**, **I435V**, **I527T** show in structure
A/Shymkent/NRL-1871/2024	-	HA A/Sydney/5/2021(H1N1)	98.361	96.996	9	**S100P**, **N111D**, **T137A**, **I183V**, **K186Q**, **A233T**, **R240Q**, **I435V**, **I527T** show in structure
A/Shymkent/NRL-1872/2024	-	HA A/Sydney/5/2021(H1N1)	98.361	96.996	9	**S100P**, **N111D**, **T137A**, **I183V**, **K186Q**, **A233T**, **R240Q**, **I435V**, **I527T** show in structure
A/Shymkent/NRL-1873/2024	-	HA A/Sydney/5/2021(H1N1)	98.361	96.996	9	**S100P**, **N111D**, **T137A**, **I183V**, **K186Q**, **A233T**, **R240Q**, **I435V**, **I527T** show in structure
A/Shymkent/NRL-1875/2024	-	HA A/Sydney/5/2021(H1N1)	97.996	96.996	11	**S100P**, **N111D**, **T137A**, **K186Q**, **V190I**, **S221W**, **R222K**, **A233T**, **R240Q**, **I435V**, **I527T** show in structure

A/Shymkent/NRL-1999/2024	-	HA A/Sydney/5/2021(H1N1)	98.543	96.996	8	**S100P**, **N111D**, **T137A**, **K186Q**, **A233T**, **R240Q**, **I435V**, **I527T** show in structure
A/Shymkent/NRL-2001/2024	-	HA A/Sydney/5/2021(H1N1)	98.361	96.996	9	**S100P**, **N111D**, **T137A**, **K186Q**, **R222K**, **A233T**, **R240Q**, **I435V**, **I527T** show in structure
A/Shymkent/NRL-2007/2024	-	HA A/Sydney/5/2021(H1N1)	98.361	96.996	9	**S100P**, **N111D**, **T137A**, **I183V**, **K186Q**, **A233T**, **R240Q**, **I435V**, **I527T** show in structure
A/Shymkent/NRL-2012/2024	-	HA A/Sydney/5/2021(H1N1)	98.361	96.996	9	**S100P**, **N111D**, **T137A**, **I183V**, **K186Q**, **A233T**, **R240Q**, **I435V**, **I527T** show in structure
A/Shymkent/NRL-2013/2024	-	HA A/Sydney/5/2021(H1N1)	98.361	96.996	9	**S100P**, **N111D**, **T137A**, **I183V**, **K186Q**, **A233T**, **R240Q**, **I435V**, **I527T** show in structure
A/Shymkent/NRL-2019/2024	-	HA A/Sydney/5/2021(H1N1)	98.361	96.996	9	**S100P**, **N111D**, **T137A**, **I183V**, **K186Q**, **A233T**, **R240Q**, **I435V**, **I527T** show in structure
A/Shymkent/NRL-2020/2024	-	HA A/Sydney/5/2021(H1N1)	96.539	96.996	19	**C84F**, **E85K**, **N101S**, **N111D**, **E120K**, **S124K**, **R130W**, **F131V**, **T137A**, **S174T**, **K186Q**, **S221Q**, **S224N**, **I231T**, **A233T**, **R234K**, **R240Q**, **K322T**, **I435V** show in structure
A/Shymkent/NRL-2021/2024	-	HA A/Sydney/5/2021(H1N1)	98.725	96.996	7	**N111D**, **T137A**, **K186Q**, **A233T**, **R240Q**, **I435V**, **G478R** show in structure
A/Shymkent/NRL-2025/2024	-	HA A/Sydney/5/2021(H1N1)	98.907	96.996	6	**N111D**, **T137A**, **K186Q**, **A233T**, **R240Q**, **I435V** show in structure

A/Shymkent/NRL-2026/2024	-	HA A/Sydney/5/2021(H1N1)	98.361	96.996	9	**S100P**, **N111D**, **T137A**, **I183V**, **K186Q**, **A233T**, **R240Q**, **I435V**, **I527T**<br>show in structure
A/Shymkent/NRL-2027/2024	-	HA A/Sydney/5/2021(H1N1)	98.543	96.996	8	**S100P**, **N111D**, **T137A**, **K186Q**, **A233T**, **R240Q**, **I435V**, **I527T**<br>show in structure
A/Shymkent/NRL-2029/2024	-	HA A/Sydney/5/2021(H1N1)	98.725	96.996	7	**N111D**, **T137A**, **K186Q**, **A233T**, **R240Q**, **I435V**, **V537A**<br>show in structure
A/Shymkent/NRL-2030/2024	-	HA A/Sydney/5/2021(H1N1)	98.907	96.996	6	**N111D**, **T137A**, **K186Q**, **A233T**, **R240Q**, **I435V**<br>show in structure
A/Shymkent/NRL-2033/2024	-	HA A/Sydney/5/2021(H1N1)	98.361	96.996	9	**S100P**, **N111D**, **T137A**, **I183V**, **K186Q**, **A233T**, **R240Q**, **I435V**, **I527T**<br>show in structure
A/Shymkent/NRL-2036/2024	-	HA A/Sydney/5/2021(H1N1)	98.361	96.996	9	**S100P**, **N111D**, **T137A**, **I183V**, **K186Q**, **A233T**, **R240Q**, **I435V**, **I527T**<br>show in structure
A/Shymkent/NRL-2037/2024	-	HA A/Sydney/5/2021(H1N1)	98.361	96.996	9	**S100P**, **N111D**, **T137A**, **I183V**, **K186Q**, **A233T**, **R240Q**, **I435V**, **I527T**<br>show in structure
A/Shymkent/NRL-2038/2024	-	HA A/Sydney/5/2021(H1N1)	98.361	96.996	9	**S100P**, **N111D**, **T137A**, **I183V**, **K186Q**, **A233T**, **R240Q**, **I435V**, **I527T**<br>show in structure

Right-click here to save/download **detailed mutation report** table for archiving or import to Excel (Tab-separated, one mutation per line)
Right-click here to save/download **query summary report** table for archiving or import to Excel (Comma-separated, one query per line)
Right-click here to save/download **query to clade call, HPAI/LPAI predictions and drug sensitivity details** table for archiving or import to Excel (Tab-separated, one query per line)

Warning: this reference selection includes sequences of strains without complete genomes, e.g. only HA and NA available/included. Therefore, hits of other segments to different reference strains do not represent reassortments. Please see also this help section regarding reassortments.

Mutations are color-coded based on their known or predicted biological significance. When there are no known effects for a mutation, it will be displayed in **black** and assigned an interest level of 0 (minimally significant). When a mutation is a common subtype marker, it will be displayed in **green** and assigned an interest level of 0 (minimally significant). Mutations occurring at the interaction site will be displayed in blue and assigned an interest level of 1 (moderately significant). If a mutation occurs at a site known to be involved in drug binding or alter host cell specificity, it will be displayed in orange and assigned an interest level of 2 (significant). Mutations will also be displayed in **orange** and assigned an interest level of 2 if the equivalent site is known to cause antigenic shifts or mild forms of drug resistance. Mutations that create or eliminate potential glycosylation sites are colored **purple** and assigned a level of interest of 2. Only mutations known to alter viral virulence, cause strong drug resistance, or divert the effects of the premature STOP codon in the PB1-F2 gene of the H1N1 pandemic appear in red and are assigned a level of interest of 3 (most significant).

https://app2.epicov.org/epi3/cfrontend#4c7d8a
