# Supplementary material for: Study of Molecular Genetic Characteristics of Influenza A(H1N1) Viruses Identified During Monitoring in Kazakhstan (2023–2026)
Source: Viruses. 2026 Oct 1;18(10):1087. doi: 10.3390/v18101087 (PMC13709524; doi:10.3390/v18101087)
Supplement: Supplementary file 1 [file viruses-18-01087-s001.zip › S2_NA_List_mutations_FluSurver.pdf]

**Table S2.** Mutations in the amino acid sequence of the NA gene of Kazakhstani strains relative to the most suitable reference A/Missouri/11/2025(H1N1)

| Query | % AA identity | % length coverage | mutations | List of mutations |
|---|---|---|---|---|
| A/Almaty/01/2025 | 99.359 | 99.787 | 3 | S52N, V98A, V234I<br>NA drug sensitivity positions: 58, 14, 0 |
| A/Shymkent/NRL-1863/2024 | 99.145 | 99.787 | 4 | I46V, S52N, V98A, K389T<br>NA drug sensitivity positions: 57, 14, 0 |
| A/Shymkent/NRL-1866/2024 | 99.359 | 99.787 | 3 | V98A, D199N, M314I<br>NA drug sensitivity positions: 57, 15, 0 |
| A/Shymkent/NRL-1868/2024 | 99.145 | 99.787 | 4 | I46V, S52N, V98A, G414E<br>NA drug sensitivity positions: 58, 14, 0 |
| A/Shymkent/NRL-1875/2024 | 99.359 | 99.787 | 3 | V98A, D199N, M314I<br>NA drug sensitivity positions: 57, 15, 0 |
| A/Shymkent/NRL-1999/2024 | 98.932 | 99.787 | 5 | S52N, A75V, V98A, I223R, V234I<br>NA drug sensitivity positions: 57, 15, 0 |
| A/Shymkent/NRL-2001/2024 | 98.932 | 99.787 | 5 | V80M, V98A, D199N, M314I, D324E<br>NA drug sensitivity positions: 57, 15, 0 |
| A/Shymkent/NRL-2020/2024 | 99.359 | 99.787 | 3 | I46V, V98A, G382R<br>NA drug sensitivity positions: 58, 14, 0 |
| A/Shymkent/NRL-2021/2024 | 99.786 | 99.787 | 1 | V98A<br>NA drug sensitivity positions: 58, 14, 0 |
| A/Shymkent/NRL-2025/2024 | 99.786 | 99.787 | 1 | V98A<br>NA drug sensitivity positions: 58, 14, 0 |
| A/Shymkent/NRL-2027/2024 | 99.359 | 99.787 | 3 | S52N, V98A, V234I<br>NA drug sensitivity positions: 58, 14, 0 |
| A/Shymkent/NRL-2029/2024 | 99.573 | 99.787 | 2 | V98A, R220K<br>NA drug sensitivity positions: 58, 14, 0 |
| A/Shymkent/NRL-2030/2024 | 99.145 | 99.787 | 4 | S52N, V98A, V234I, S247N<br>NA drug sensitivity positions: 57, 15, 0 |

| | | | | |
|---|---|---|---|---|
| A/Shymkent/NRL-2036/2024 | 99.359 | 99.787 | 3 | **I46V**, **S52N**, **V98A**<br>NA drug sensitivity positions: **58, 14, 0** |
